# Wetting mechanism and morphological adaptation; leaf rolling enhancing atmospheric water acquisition in wheat crop—a review

**DOI:** 10.1007/s11356-022-18846-3

**Published:** 2022-02-01

**Authors:** Zulfiqar Ali, Sabah Merrium, Muhammad Habib-ur-Rahman, Sadia Hakeem, Muhammad Abu Bakar Saddique, Muhammad Ali Sher

**Affiliations:** 1 Institute of Plant Breeding and Biotechnology, MNS-University of Agriculture, Multan, 60000 Pakistan; 2 Institute of Crop Science and Resource Conservation (INRES), Crop Science Group, University of Bonn, Bonn, Germany; 3 Department of Agronomy, MNS-University of Agriculture, Multan, 60000 Pakistan

**Keywords:** Morphological adaptations, Atmospheric gaseous water harvesting, Contact angle hysteresis, Drought

## Abstract

Several plant species such as grasses are dominant in many habitats including arid and semi-arid areas. These species survive in these regions by developing exclusive structures, which helps in the collection of atmospheric water. Before the collected water evaporates, these structures have unique canopy structure for water transportation that plays an equivalent share in the fog-harvesting mechanism. In this review, the atmospheric gaseous water harvesting mechanisms and their affinity of measurements were discussed. Morphological adaptations and their role in the capturing of atmospheric gaseous water of various species were also discussed. The key factor for the water collection and its conduction in the wheat plant is the information of contact angle hysteresis. In wheat, leaf rolling and its association with wetting property help the plant in water retention. Morphological adaptations, i.e., leaf erectness, grooves, and prickle hairs, also help in the collection and acquisition of water droplets by stem flows in directional guide toward the base of the plant and allow its rapid uptake. Morphological adaptation strengthens the harvesting mechanism by preventing the loss of water through shattering. Thus, wheat canopy architecture can be modified to harvest the atmospheric water and directional movement of water towards the root zone for self-irrigation. Moreover, these morphological adaptations are also linked with drought avoidance and corresponding physiological processes to resist water stress. The combination of these traits together with water use efficiency in wheat contributes to a highly efficient atmospheric water harvesting system that enables the wheat plants to reduce the cost of production. It also increases the yielding potential of the crop in arid and semi-arid environments. Further investigating the ecophysiology and molecular pathways of these morphological adaptations in wheat may have significant applications in varying climatic scenarios.

## Introduction

Wheat (*Triticum aestivum* L.) is of major dietary importance worldwide. It is a valuable source of major nutrients that can reduce the risk of human diseases such as diabetes type II, colon or rectal cancer, and cardiovascular disease (Shewry and Hey 2015). Global estimated wheat production reported 730.7 million tons in 2018–2019. While it is projected that the global population may reach 9.1 billion by 2050, there is a need to produce 70% more wheat to fulfill the demands of an ever-increasing global population to ensure food security (FAO et al. 2019). Ray et al. (2013) reported that global wheat production is 38% less than projected demand in the future (2050). This reduction may be compromised by several factors such as climate change, insufficient freshwater, and lower resource use efficiency. For its development and production, a wheat crop is entirely dependent on the weather and other environmental conditions. Climatic models predict more anomalies such as elevated temperature and drought events in near future (Martens 2013; Amin et al. 2018; Rahman et al. 2018; Saddique et al. 2020a). Among the many negative impacts of climate change, rising ambient temperatures are considered to be the most detrimental to cereal crops including wheat crop (Ahmad et al. 2015; Yasin et al. 2021; Hussain et al. 2021; Saddique et al. 2020b,c). Demirhan (2020) reported that a 1 °C increase in temperature will significantly reduce global wheat production. Currently, water is being consumed via irrigation, manufacturing industries, thermal electric power, domestic household use, mining, aquaculture, and general public supply. Agriculture alone consumes 92% (25,000 km^3^ year^−1^) of rain and flowing water for crops, livestock, fisheries, and forestry (Appelgren 2004; Sajid and Rahman 2021). Atmospheric water is available on earth in the form of rainfall, snowfall, foggy clouds, dew, mist, and humidity vapors. It contributes 0.001% of global water that is equal to 3.3% of freshwater (Bhushan 2019).

Climate changes, increased human growth and demand, unsustainable agricultural practices, and deforestation negatively affect the worldwide accessibility of freshwater (Trenberth et al. 2014). Among these stressors, global warming and long periods of drought cause the most significant decline in freshwater supply (Bhushan 2020). The intensified effects of predicted changes in climate and global population growth will significantly raise the demand for freshwater. The United Nations estimates that five billion people may suffer from water shortages by 2050 (Trenberth et al. 2014). So, there is a need to supplement the current supply of freshwater to satisfy the demands of the future.

Drought can affect all phenological stages of wheat and can have negative impacts on morphological, physiological, and biochemical traits (Nasim et al. 2018; Ali 2019; Ul Hassan et al. 2021). Leaves are the main organs of photosynthesis. Drought can negatively affect the leaves and cause a reduction in the assimilation process, leaf expansion, stomatal conductance, and this can lead to a decrease in yield. To compensate for the adverse effects of drought stress, several morphological adaptations such as leaf rolling and leaf erectness have been reported in the wheat plant due to loss of turgor pressure and osmotic adjustment (Mossa et al. 2016). Leaf rolling is a common adaptive response to drought stress in plants. Leaf rolling is caused by a change in the water potential within the epidermal and bulliform cells. Leaf rolling slows down transpiration and enhances the accumulation of dry matter (Lang et al. 2004). Leaf rolling dynamics such as inward or outward rolling also allow efficient photosynthetic activities in leaves (Yuan et al. 2015). Optimum expression of leaf rolling is effective for the increase in water use efficiency instead of completely rolled leaf dynamics (Juarez et al. 2004). Similarly, leaf rolling in wheat reduces the energy load on the leaf, lowering the surface temperature of the leaf, allowing at the same time the light to go deeper into the canopy, thus improving light interception and also reducing water loss (Rebetzke et al. 2001). In wheat, genotypic differences have been reported for leaf rolling but studies related to the genotypic variation of leaf rolling are rare (Sirault et al. 2015).

Living organisms may provide the solution to uncovering new resources of fresh available water. Many plant and animal species survive by exhibiting efficient approaches/techniques of water harvesting/collection from the atmosphere in arid regions. These solutions involve leaf surface structure in plants and their chemistry with atmospheric water. These unique surface structures of different species help in the directional pathways of water or storage within their body tissues (Brown and Bhushan 2016). Many scientists have been working together to save groundwater by the development of innovative bio-inspired water harvesters. Some desert species such as cactus, grasses, and bushes intercept the atmospheric water by using their external surface structures and chemistry combination to transport to areas where it can be stored and consumed. Association of morphological adaptations with wetting events such as fog drip and stem flow alters the hydrological conditions of the plant (Roth-Nebelsick et al. 2012). Fog and dew enhance leaf turgor, plant growth, and also photosynthetic pathways (Xu et al. 2018).

Many plant species use fog as an additional water supply. For its acquisition, two possibilities exist in plants during leaf-wetting events. Plants may gain access to fog water through root uptake of coalesced water that drips to the soil or by direct foliar uptake of water retained by the crowns of the plant. Leaf rolling is a beneficial trait in wheat that can help to move atmospheric water towards the root zone similar to interception of fog water by spiral leaf rolling of *Stipagrostis sabulicola* in Namib Desert (Roth-Nebelsick et al. 2012). Bulliform cells (large specialized adaxial epidermal cells) responsible for leaf rolling also help a wheat plant to increase wettability. Therefore, leaf rolling dynamics of a wheat plant supports the high input use efficiency that can compensate yield losses under drought stress.

Atmospheric water is an efficient water source for plants which will be discussed in the next sections. Crops that are grown in the foggy months can be architected to harvest atmospheric water for irrigation purposes. Wheat plant canopy can be designed and modified in such a way that intercept the atmospheric water and also enhance the water use efficiency and ultimately yield. The genetic potential of the wheat plant should also be exploited by wheat breeders to enhance the yielding potential. There is a need to conduct studies to exploit the genetic diversity of structural adaptations and their chemistry with the wetting mechanisms for harvesting atmospheric water.

This review summarizes the water availability and the impact of water shortages on wheat growth, but particularly emphasizes on the innovative strategy to overcome the water shortage by using fog water. The first part of the review focuses on the effects and responses of drought stress on wheat crop and the second part discussed the potential of leaf rolling dynamics in fog capturing. The section that follows discusses the leaf-wetting mechanism and its potential in different species. Collection and retention of fog water in wheat through morphological adaptation, i.e., leaf rolling, longitudinal grooves, leaf erectness, and prickle hairs, will also be discussed.

## Water availability

**Fig. 1:**
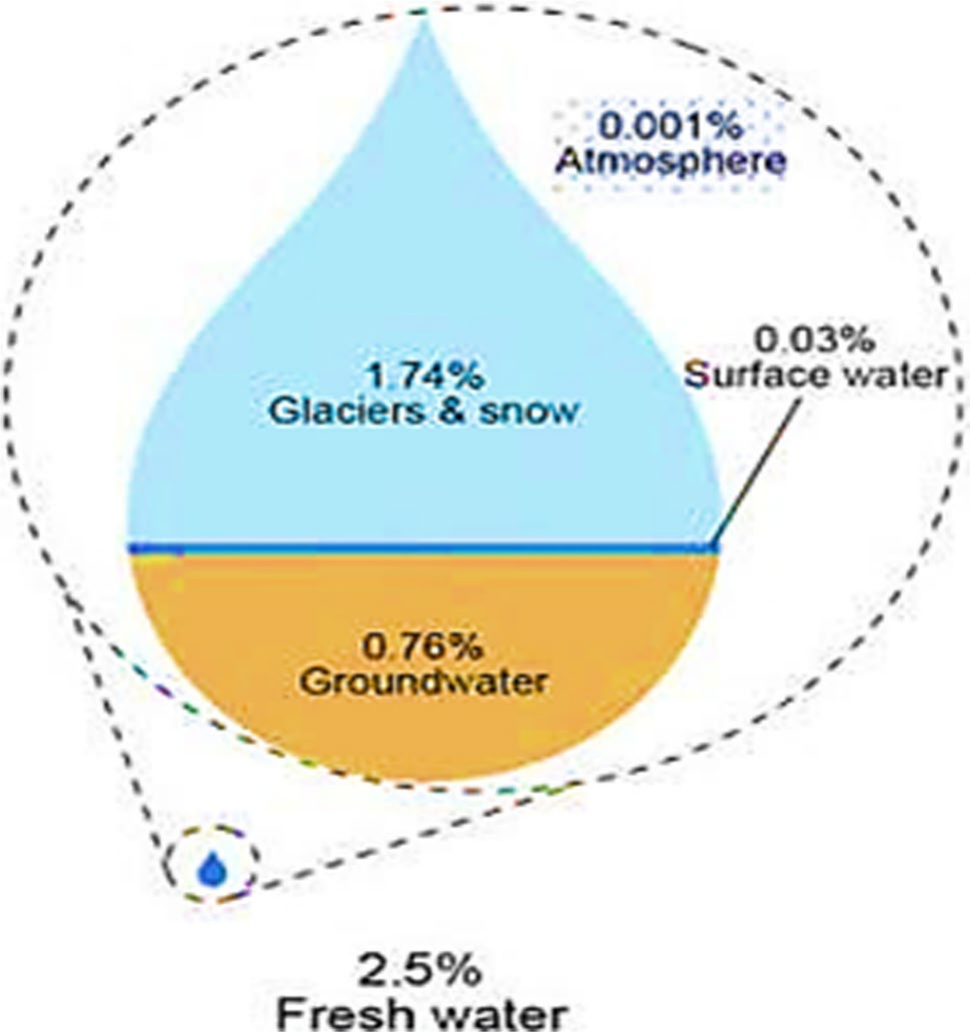
Breakdown of freshwater (Shiklomanov 1993)

Earth has an abundance of water and is known as a blue planet because 70% of the earth’s surface is covered by water (Brown and Bhushan 2016; Bhushan 2020). The source of freshwater utilized for drinking purposes is 2.5% only (Fig. 1). A large portion of freshwater is trapped in glaciers and snow while 0.26% is available for human consumption. Water required by a person is 4 l while 2000 l is required for food production. Forty percent of the world’s grain harvest comes from irrigated lands (World Bank 2020). Over-pumping and urbanization put pressure on the water-based food production system (Bhushan 2016, 2019, 2020). As a result, there is a need to minimize the usage of freshwater resources in agricultural production systems, and to satisfy the water requirement in growing economic sectors. An important goal is to decrease water consumption and to increase the water use efficiency of crop production by developing water-saving techniques.

## Growth and development of phenological phases of a wheat crop

**Fig. 2:**
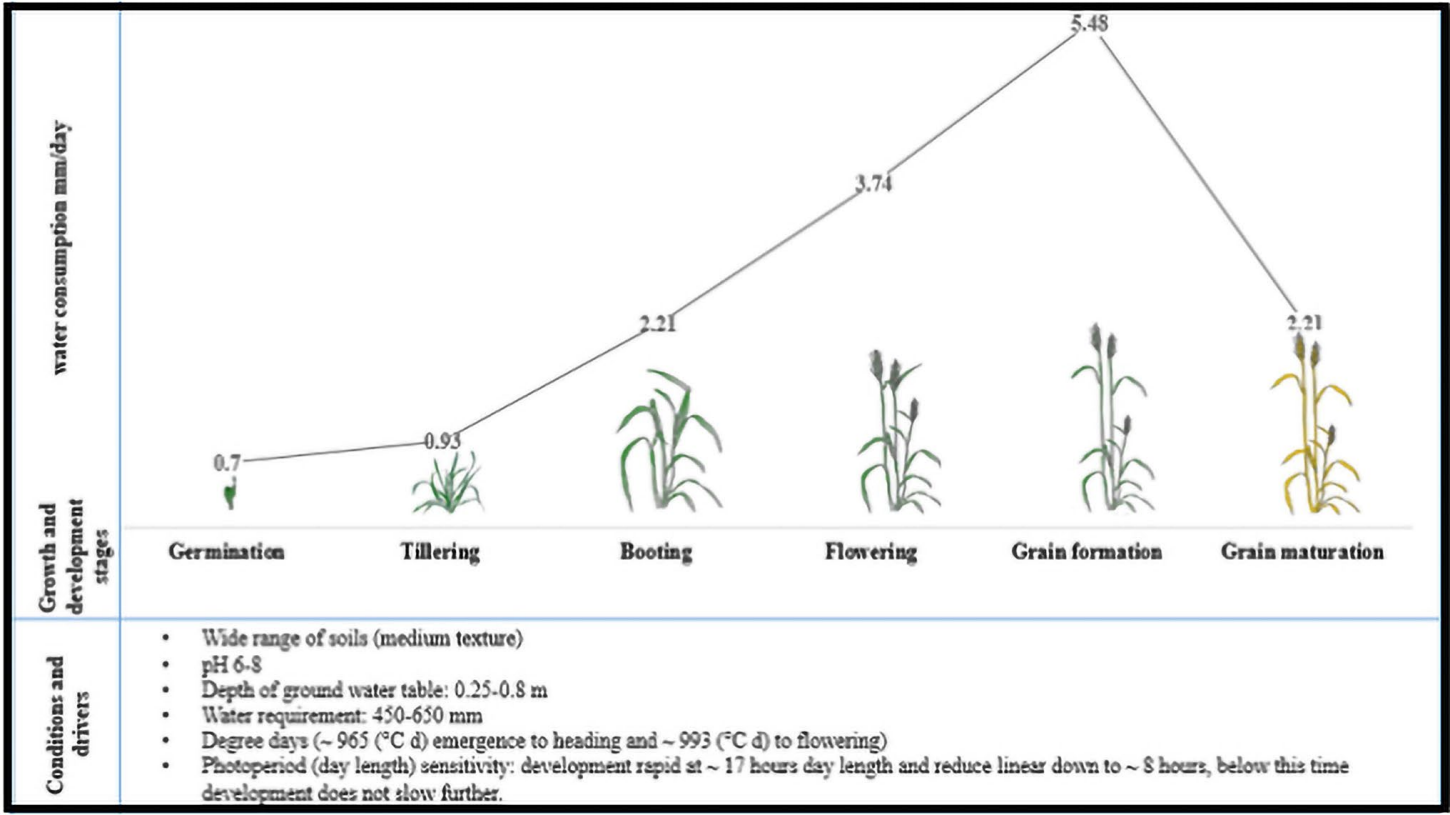
Daily water consumption of wheat plants at different growth and development stages

Growth and development are two major drivers of wheat that contribute to optimal production and yield. Both are different in their functions but dependent on each other. Growth relates to the accumulation of dry matter by photosynthesis and radiation efficiency while development relates to the speed at which wheat moves through its life cycle (Porter and Gawith 1999). A plant has five major phenological phases of development (emergence to ripening), and its synchronization with environmental conditions is essential to optimize the production of biomass and yield (Flohr et al. 2018). Environmental conditions such as water, temperature, solar radiation, and day length are major drivers of the development cycle (Rahman et al. 2021). Among all the drivers, water is the most significant and its prerequisite relies upon the stage of development, weather conditions, and different types of soil. Water requirement varies from emergence to maturity and the conditions required for development at these growth stages are shown in Fig. 2 (Bloomfield et al. 2018; Hyles et al. 2020).

Uptake of water, nutrients, and CO_2_ increases from germination to maturity and a plant uses these for energy requirements, photosynthesis, respiration, evapotranspiration, and development (Blum 2011). Low water availability/supply for long periods during the developmental cycle can reduce the number of days and affect the physiological processes (photosynthetic rate and stomatal conductance) that cause the disturbance of the enzymes and inflow of CO_2_ (Centritto et al. 2009; Akram et al. 2013). It can also affect water relations, canopy temperature, and rate of transpiration (Farooq et al. 2013). Excessive water supply either in the form of groundwater or rainfall also leads to a reduction in grain quality and germination of grains in the ear (crop maturity phase), and losses in grain yield (Guarienti et al. 2005).

## Effect of a shortage of water at different phenological phases of growth

Low water supply causes drought stress conditions during crop growth and its development cycle. Meta-analysis of wheat revealed that a water shortage at different phenological phases causes a 27.5% reduction in yield (Zhang et al. 2018). The intensity and frequency of a water shortage at different phenological phases are critical to the growth and production of a wheat crop (Sarto et al. 2017). Wheat plants may be more susceptible to drought at specific critical growth stages. At germination and seedling stages, traits that are more susceptible to drought stress include seedling vigor, coleoptile length, and root/shoot ratio (Kızılgeçi et al. 2017). These traits caused a 7% yield reduction under moderate stress levels, but its recovery was rapid after re-watering (Zhang et al. 2013). Studies related to the seedling stage revealed a positive association of these traits related to yield (Dodig et al. 2015). At tillering and stem elongation, severe drought reduces the number of spikelets and the number of grains that eventually cause a reduction in grain yield. For example, Abid et al. (2018) observed a yield reduction of 6–16% at tillering and 15–24% at stem elongation under severe drought stress while at stem elongation, a 72% reduction in yield was observed.

The source-to-sink relationship of a plant is also affected and this results in the accumulation of more carbon in the roots than the shoots. This has been known to cause a reduction in plant biomass (Ding et al. 2018). The heading and flowering stage is most susceptible to drought and causes multiple impacts such as a decrease in the number of grains per spike and the overall spike weight. Evapotranspiration is maximized at heading and flowering under stress which aggravates the condition and causes even more severe losses. Due to severe drought stress, 46–82% yield reduction has been reported (Khakwani et al. 2012) while only 19–42% under moderate levels (Maqbool et al. 2015). The grain filling stage is also severely affected by drought stress due to the lack of recovery time compared to other growth stages. Drought stress harms the photosynthetic assimilation during the grain filling stage and results in a 5.2%, 20.7%, and 28% decline in kernel weight, grain numbers, and yield, respectively (Gevrek and Atasoy 2012). Damages from drought stress at different phenological phases are unrecoverable.

To neutralize the drought’s adverse consequences, a plant normally adopts three mechanisms, i.e., escape, tolerance, and avoidance (Gilbert and Medina 2016). The escape mechanism allows a plant to rapidly complete its life cycle before the onset of stress. As a result of this stress, the plant will produce fewer seeds. The drought tolerance mechanism comprises low tissue water potential and osmotic adjustments which help the plant to maintain turgor. The drought avoidance mechanism allows the plant to maintain water potential, grow deeper roots in the soil, increase its transpiration rate, and decrease the amount of water extracted from tissues (Blum 2011).

## Responses of a wheat to drought stress

**Fig. 3:**
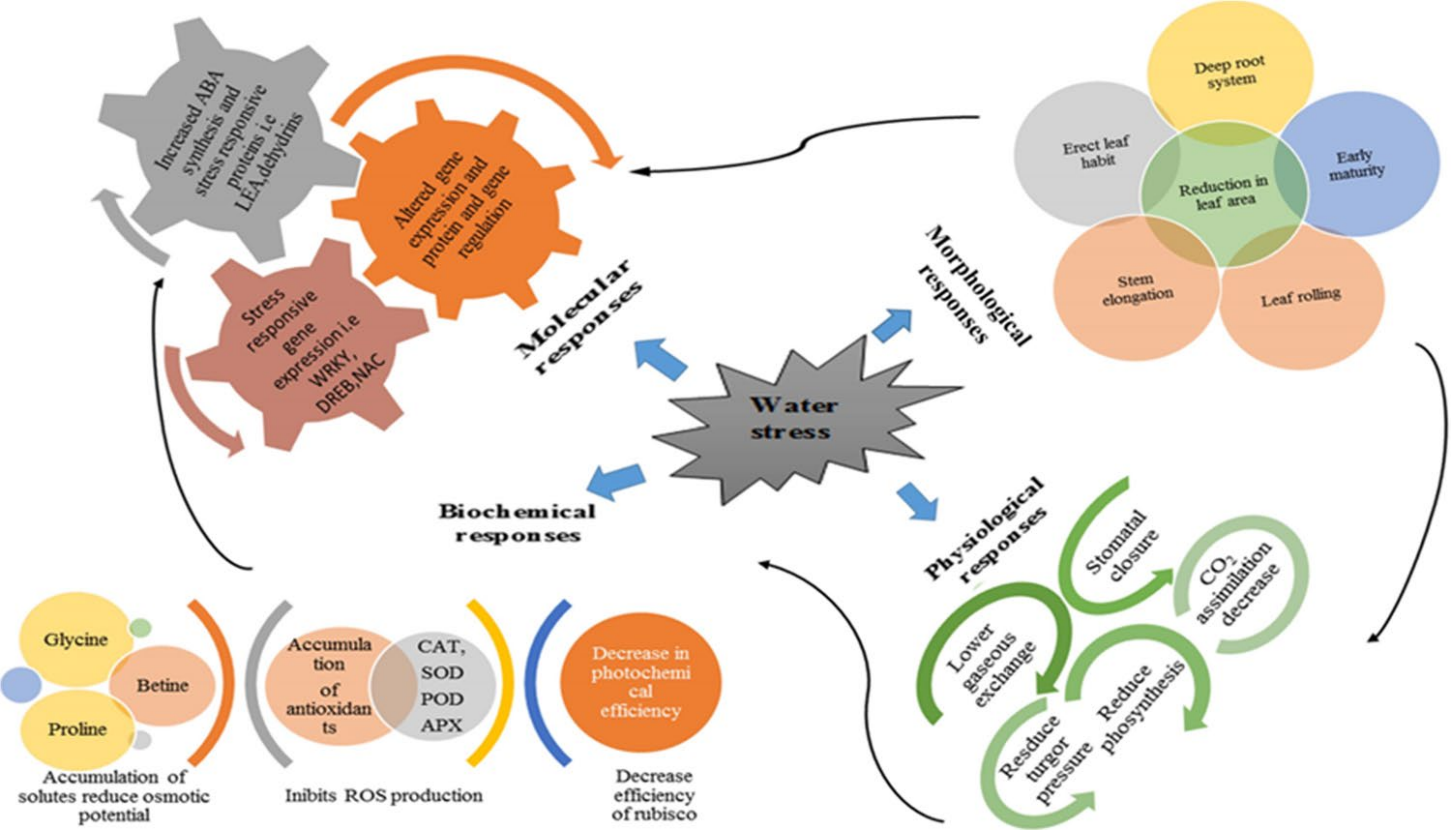
Wheat plant responses to water stress by complex mechanisms

Adaptation of a plant to drought stress is an emerging issue. Many factors can affect the responses of drought stress such as physiological processes, intensity and frequency of stress, growth stage, and environmental factors (Blum 2011). When water stress is sensed by a plant during phenological development, this leads to the emergence of different responses to help the plant survive under stressful conditions (Fig. 3).

### Morphological response

Drought stress affects various morphological traits of a wheat plant. It results in poor root development and leaf growth characteristics including shape, size, leaf area, intensity, composition of cuticle waxiness and pubescence, dry weight, density, and length of roots (Nezhadahmadi et al. 2013). Plant’s responses to drought stress can be manifested by a deep root system, leaf rolling, early maturity, erect leaf habit, and reduced leaf area can be associated to drought tolerance (Fig. 3) (Kahlown et al. 2007; Kadioglu and Terzi 2007; Nezhadahmadi et al. 2013). Wheat plant under water stress rolls up their leaves due to loss of turgor pressure, this condition is called wilting. This condition mostly happens during the hottest part of the day due to the high rate of transpiration. Re-expansion of the rolled leaves occurs gradually at night time when transpiration rate is lower than that of the day time (Araus et al. 2008). Under water stress, leaf rolling and the upright posture of leaves reduce the energy load on the leaf which lowers the surface temperature of the leaf, allowing the light to penetrate deeper into the canopy, which improves light interception and also reduces water loss (Rebetzke et al. 2001).

The presence of trichomes, their density, and waxiness on leaves may reduce the loss of water and act as a protective shield against water stress for longer periods (Bowne et al. 2012). Yield stability under drought stress is crucial. Moderate leaf rolling maximizes photosynthesis and contributes to more yield potential under drought conditions (Zhang et al. 2009a). Leaf rolling and erect leaf angle in plants prevent leaves from photo-damage, reduce transpiration by reducing leaf area, and boost water use efficiency (Kahlown et al. 2007; Kadioglu and Terzi 2007). Under drought stress, most affected organ of the plant is the root system. Its growth continues in search of water and forms a prolific deep-rooted system to facilitate water uptake (Comas et al. 2013). Under drought stress, a wheat plant also shows early maturity, reduction in plant size and leaf area for the equilibrium of water by the root system and plant tissues (Liwani et al. 2019).

### Physiological and biochemical responses

Drought stress also negatively affects various physiological responses in wheat such as gaseous exchange, leaf water potential, and relative water content (Nezhadahmadi et al. 2013; Huseynova et al. 2016). Root-shoot ratio is also regulated with the concentration of abscisic acid to enhance water absorption under drought stress (Mahdid et al. 2011; Nezhadahmadi et al. 2013). Abscisic acid (ABA) controls plant growth by modifying the development of leaves and roots (Farooq et al. 2013; Reddy et al. 2014). The low water status of plants causes the closure of stomata and reduces the photorespiration rate, which as a result improves water use efficiency (Keyvan 2010). Leaf chlorophyll content also reduces under water stress but its intensity of reduction depends on the age of the leaves. In wheat, younger leaves have more chlorophyll content due to the activation of enzymes but older leaves show a reduction (13–15%) due to the inactivation of enzymes (Nikolaeva et al. 2010). The accumulation of osmolytes under stress allows cells to maintain their integrity and cellular dehydration (Loutfy et al. 2012). Under water stress conditions, wheat accumulates several inorganic (potassium, calcium, silicon, and salicylic acid) and organic solutes (glycine betaine, proline, soluble carbohydrates, and proteins) in its cytosol for maintenance of cell turgor by lowering its osmotic potential (Pei et al. 2010; Marcińska et al. 2013; Kang et al. 2013; Gou et al. 2015).

In wheat plants, the increased production of reactive oxygen species (ROS) results in alterations of cellular structure and negatively affects photosynthesis. They also cause the inactivation of enzyme activities and oxidative damages (Miller et al. 2010; Huseynova et al. 2016). In response to water stress, the wheat plant produces several antioxidant enzymes such as catalase (CAT), superoxide dismutase (SOD), peroxidase (POD), ascorbate peroxidase (APX), and glutathione peroxidase (GPx) that inhibit several ROS production (Gill and Tuteja 2010).

### Molecular responses of a wheat plant

Many genes and their signaling events are implicated in a wheat plant’s response to water stress. High throughput molecular studies have presented many water stress–responsive genes and their regulation is divided into two categories:

1. Functional proteins that are involved in the protection of plants from the effects of dehydration are late embryogenesis abundant (LEA) proteins and antioxidants. For the uptake and transport of water, aquaporins and sugar transporters are involved.
2. Regulatory proteins (kinase protein, phosphatase, and transcriptional regulatory factors—APETALA2/ethylene-responsive element-binding proteins (AP2/EREBP), zinc finger protein (bZIP), and myeloblastosis oncogene protein (MYB)) are signaling cascades that are involved in the induction of transcriptional factors and activation of targeted genes (Zahoor et al. 2020)

In wheat, these functional and regulatory proteins are involved in avoidance and tolerance mechanisms by providing osmoprotectant, osmotic adjustment, antioxidant defense system, and scavenging of oxygen radicals abilities (Yang et al. 2010; Ashraf 2010). The transcription regulation mechanism is divided into two pathways, ABA-dependent and ABA-independent (Budak et al. 2013). ABA-dependent pathways arbitrate stress adjustment by induction of two transcriptional factors (TFs): (1) zinc finger proteins (bZIP), (2) MYC (myelocytomatosis oncogene)/MYB (myeloblastosis oncogene) (Cramer et al. 2011). ABA-independent pathway of regulon includes CBF/DREB (cold-binding factor/dehydration-responsive element binding), NAC, and ZF-HD (zinc finger homeodomain) (Lata and Prasad 2011).

Both pathways were regulated by several transcriptional factors (TFs). Transcriptional factors, WRKY (TFs), were controlling various processes under stress conditions and are dependent on the ABA hormone for its regulation. In wheat, *WRKY1* and *WRKY2* transcriptional factors were up-regulated under water stress (Proietti et al. 2011). Similarly, in drought-tolerant wheat, 35 WRKY transcripts belonging to 10 *TaWKRY* genes were detected and its expression was up-regulated in the leaf and root-shoot tissues (Okay et al. 2014). NAC (TFs) also play important role in plant development under abiotic stress responses (Nakashima et al. 2014). NAC (TFs) belonging to the TaNAC69 gene in wheat was detected and its overexpression can enhance stress tolerance (David et al. 2016). Transcriptional factors of bZIP are also termed AREB (ABA-responsive element-binding protein). *WLIP 19*, *TaOBF1*, and *TaABF1* (bZIP) TFs were also found in response to water stress which are up-regulated in response to ABA in wheat (Rahaie et al. 2013).

## Adaptations to minimize water shortage for wheat

### Leaf-wetting mechanism and characterization for atmospheric water acquisition/collection

**Fig. 4:**
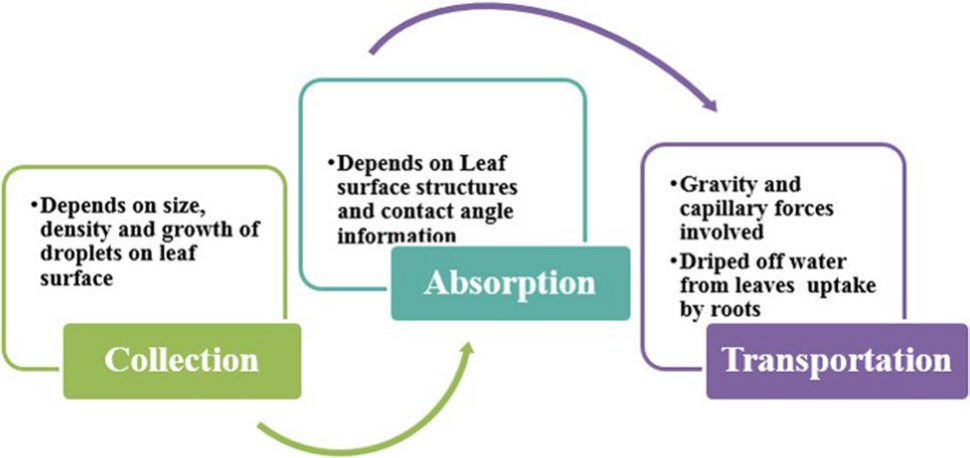
Leaf wettability mechanism by atmospheric water. An explanation of the basic processes involved in the collection of water on the leaf surface, absorption by the leaf surface, and transport of excess water to the root system of a wheat plant

The leaf surface of many plant species is frequently wetted by atmospheric water resources such as rainfall, dew, foggy clouds, mist, and haze. Water droplets are collected on both the adaxial and abaxial leaf surfaces before coalescing and moving down toward the root zone (Bhushan 2016, 2019, 2020). Leaf features such as conical shape, grooves, ridges, tiny hairs, and barbs facilitate the directional transportation of water droplets due to forces acting on them. These leaf features also have water-storing tissues that help in the absorption of water droplets called foliar water uptake. In simple words, it is concluded that atmospheric water acquisition consisted of three main processes, i.e., collection, absorption, and transportation. These processes depend on each other for full utilization of water. The collection of atmospheric water on the leaf surface is enhanced by the size, density, and growth of droplets by using the leaf features also called collecting depots that are also involved in the nucleation/transportation of droplets. Absorption of droplets by leaf features, called foliar water uptake, was characterized by contact angle information (hydrophobic and hydrophilic properties) and developmental features. Transportation/stem flow takes place due to the forces acting on the droplets and leaf surface, i.e., gravity and capillary forces. The amount of water that pools and is not absorbed by the leaf structure drips from the leaf tips and stem and is taken up by the roots (Israelachvili 1992; De Gennes et al. 2013). The mechanism of leaf wetting consists of three main cyclic processes shown in Fig. 4.

**Fig. 5:**
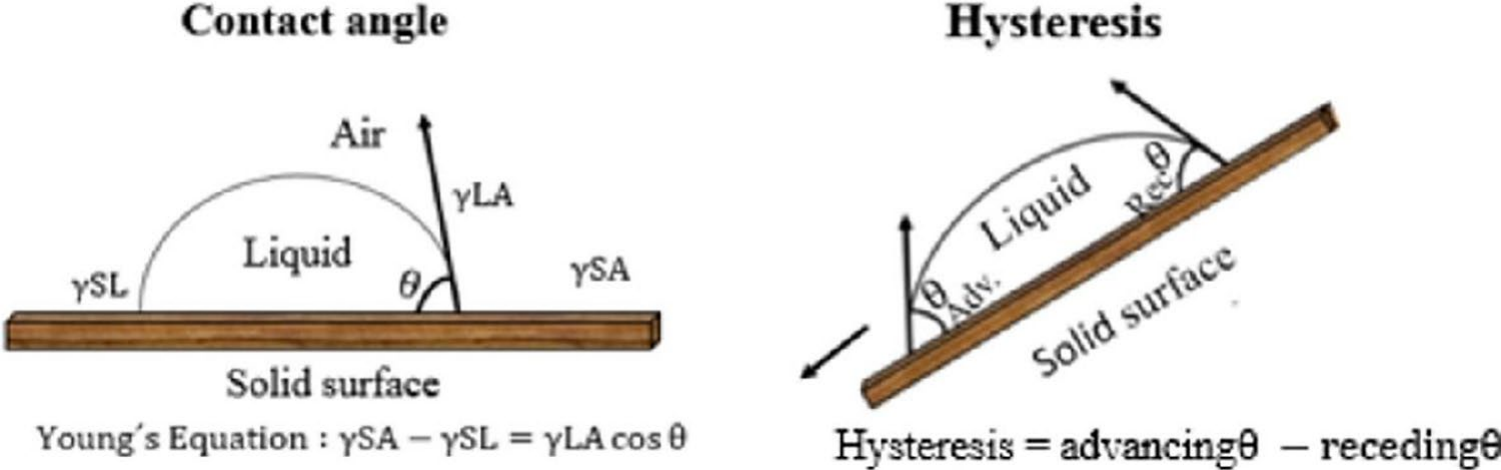
The schematic diagram shows the wetting of a solid surface. Contact angle (CA) of water droplet is described by Young’s equation (_ˠ_*LA*, _ˠ_*SL*, and _ˠ_*SA* show interfacial tensions at the boundaries between liquid (L), solid (S), and air (A)). The hysteresis of a water droplet on a tilted solid surface can be determined by measuring the advancing and receding angle of a water droplet (adapted from Barthlott et al. (2017)

The affinity of water on the leaf surface was characterized by a phenomenon called leaf wettability. The wettability of a leaf surface can be described as the process of solid–liquid interaction between two inter molecular forces interacting with each other. The degree of wettability of the leaf is measured by the contact angle (CA). The wetting behavior of a solid surface is divided into four groups based on contact angle (CA). Low contact angle values between ~ 0 and < 90° tend to spread water (hydrophilic surface) across a greater area. High contact angle values between > 90 and > 150^o^ tend to repel water (hydrophobic surface) by forming a more spherical shape (Fig. 5.) (Wenzel 1936; Wang et al. 2015; Barthlott et al. 2017).

The surface of the plant species is heterogeneous and rough. Wenzel (1936), Cassie, and Baxter (1944) established the basis for studying wetting on rough surfaces many years ago (Wenzel 1936; Cassie and Baxter 1944; Wang et al. 2015). Hysteresis (CAH) is measured by the advancing and receding angle of a moving droplet, which is responsible for sticking droplets to the leaf surface (CAH = CA_adv_. – CA_rec_,). When a droplet moves over a leaf surface, advancing CA is greater than receding CA as shown in Fig. 5. CAH is small when a droplet rolls on the solid surface with little resistance (Cassie and Baxter 1944; Barthlott et al. 2017).

**Fig. 6:**
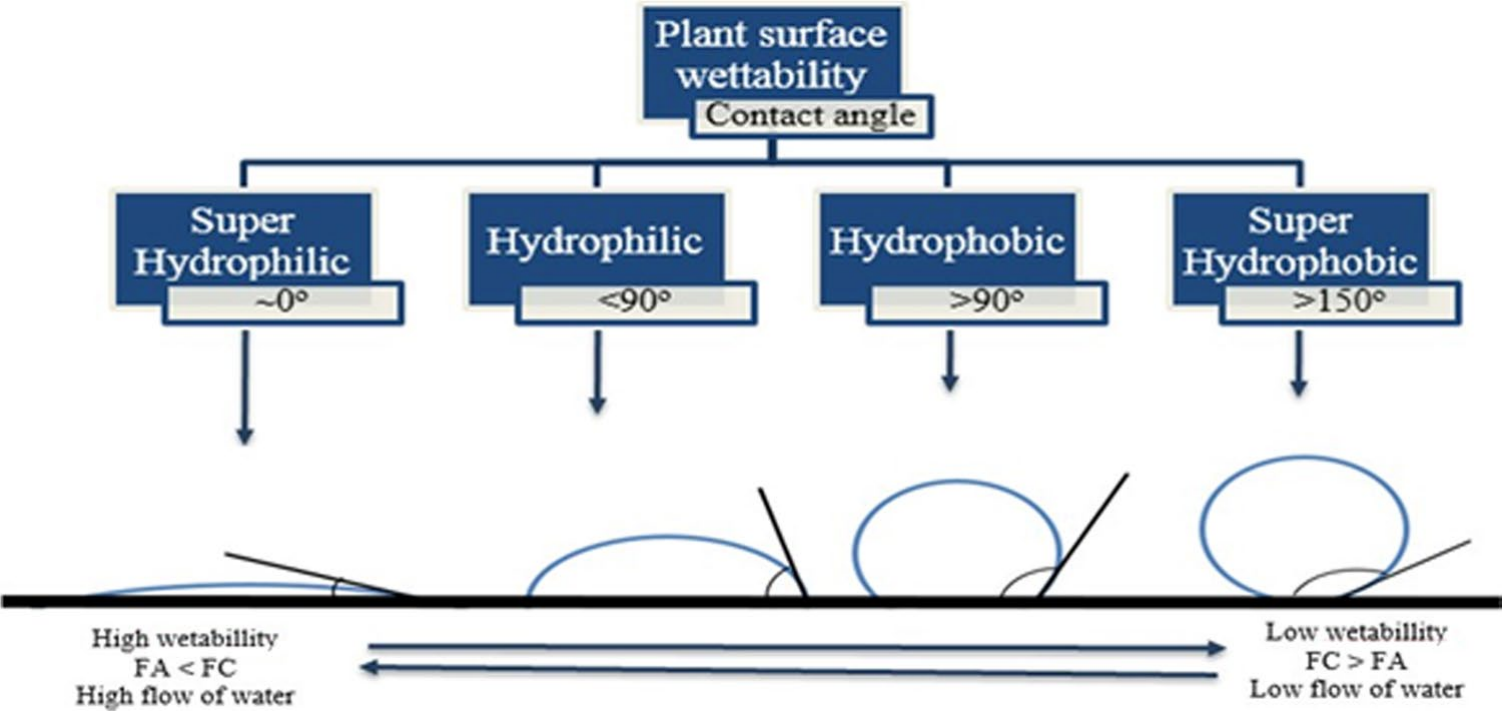
The schematic shows the characterization of wettability and contact angle of solid and liquid. FA stands for adhesive force and FC for cohesive force

In plants, super-hydrophilicity means water imposed on the plant canopy spread instantly due to gravitational forces and their surface structure chemistry. Functional attributes of these plant species include glands, hairs, and porous cells that make the plant surface hydrophilic with a high flow of water droplets. Hydrophobic surfaces have cuticular folds and dense wax deposition that helps the surface to hold the water droplet more efficiently and the flow of water is also low (Barthlott et al. 2017). When a surface is free of energy, water can move against gravity (Fig. 6).

### Atmospheric/fog water as a potential source of water for plant growth

Some recent studies have reported that, if plants have adaptive functional attributes, the acquisition and maintenance of water in their tissues could be facilitated. For its acquisition, two possibilities exist in plants during leaf-wetting events (Roth-Nebelsick et al. 2012; Rosado and Holder 2013; Eller et al. 2013; Malik et al. 2014; Berry et al. 2019). Plants may gain access to atmospheric moisture through foliar water uptake and by roots (Malik et al. 2014). Morphological adaptation and its association with wetting mechanism alleviates the drought effect, enhances water use efficiency, and increases plant growth (Roth-Nebelsick et al. 2012; Eller et al. 2013; Gürsoy et al. 2017a).

**Fig. 7:**
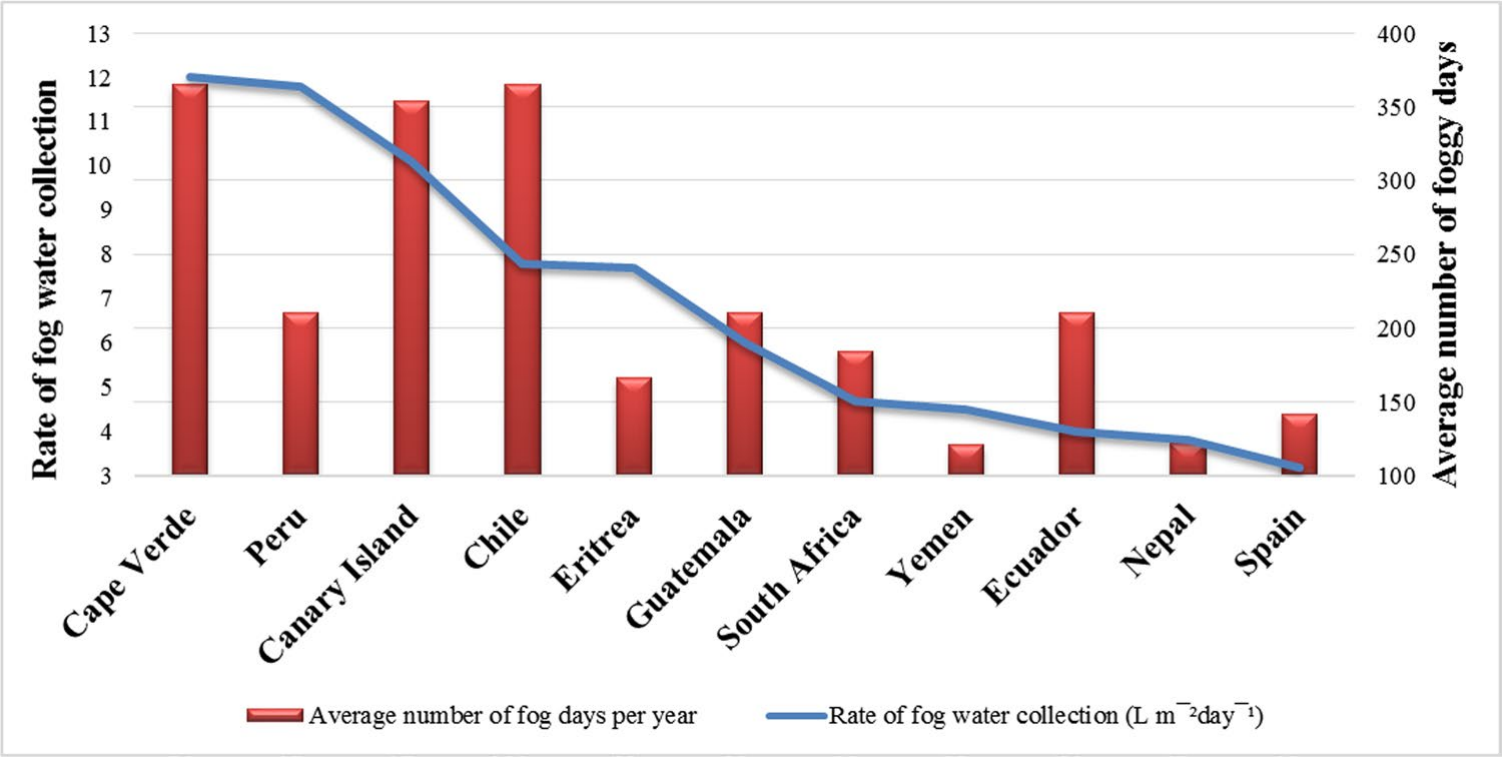
Rate of fog water collection and annual occurrence of foggy days in various countries (data source from Fessehaye et al. 2014)

Freshwater available on the earth in the form of fog, clouds, mist, and water vapors is about 0.001% (Gleick 1993; Bhushan 2016). In global terms, this quantity is very small and only accounts for 3.3% of water in rivers and lakes (Bhushan 2020). In arid regions, species survived by using these sources of water. The annual occurrence of several foggy days combined with the rate of the collection is presented in Fig. 7. In these regions, many species use fog as an additional water supply. Leaf traits of these species act as fog/atmospheric water collectors that alleviate the water crisis. This type of water collection has attained increasing interest during recent years as it is deemed to be a sustainable water resource (Brown and Bhushan 2016; Bhushan 2016, 2019, 2020). Specific details of plant species that irrigate themselves by collecting and utilizing atmospheric water are given in Table 1.

**Table 1:** Studies related to surface characteristics and water harvesting mechanisms of various plant species from atmospheric water

Sr. No	Plant	Surface characteristics and water harvesting mechanism	References
1	Herbaceous fern, *Dryopteris marginata*	Multi-scale channels or network of microscopic channels and semi-circular grooves on leaves helps to spread and transport water efficiently. Semi-circular grooves and micro-channels make the leaf surface hydrophilic with CA = 39° and CAH = 15°	Sharma et al. (2018)
2	Endemic grass species *Stipagrostis sabulicola*	Rolled leaves with longitudinal grooves and glabrous culms help in the collection and channeling of water. Due to air-driven forces and wind velocity, water droplets move horizontally along the leaf and stalk and coalesce at the base of the plant where water is taken up by the root via rhizo-sheaths CAH = 77°	Roth-Nebelsick et al. (2012)
3	Grass *Setaria viridis*	Grooves and conical shapes directionally transport water droplets	Xue et al. (2014)
4	*Cotula fallax* leaf	Fine hairs help to underpin the collection and retention of water droplets	Andrews et al. (2011)
5	Desert plant *Opuntia microdasys* (Cactus spp.)	Clusters of trichomes and conical spines on the stem of most cacti help in capturing atmospheric water while the grooves of the spine help in the movement of larger droplets which are adsorbed through trichomes at the base of spines	Kim et al. (2017)
6	*Salsola crassa*	Hair-like structures and inward rolled leaves help in the water collection mechanism	Gürsoy et al. (2017a)
7	Common shrub *Combretum leprosum Mart*	Translucent and shiny trichomes help in the collection of water droplets and their transportation. CA = 75° and 71.1° on adaxial and abaxial surfaces	Pina et al. (2016)
8	Moss (*Syntrichia caninervis*)	Barbs, also known as collections depots, aid in the collection of water. Droplets must grow large enough for movement towards the base	Pan et al. (2016)
9	Creosote bush (*Larrea tridentate*) and pencil bush (*Arthraerua leubnitziae*)	Tiny hairs help to capture water droplets, and when they get too heavy, they are redirected toward the roots. The surface structure of these species has not been studied yet	Harris and Parker (2003)

### Leaf rolling dynamics as a supplement of water in wheat

In this section, we describe the leaf rolling dynamics that are associated with drought tolerance and also have the potential to capture atmospheric water in wheat.

#### Leaf rolling dynamics

Leaf rolling is a heterogeneous trait because its expression depends on the evaporative demand and heterogeneity of soil water content. Leaf rolling is influenced by the climate and therefore has a diurnal pattern. At dawn, leaves unroll, roll at midday, and then unroll during the afternoon when there is a decrease in solar radiation and air vapor pressure deficit (Juarez et al. 2004; Ben-Amar et al. 2020). The diurnal pattern of leaf rolling reveals that it is a transient state that depends on the sensing of available moisture in the environment (Rebetzke et al. 2001).

Wheat and rice express a convolute type of leaf rolling where one side of the leaf wraps over the other resembling a twisted state. The leaf rolling trait is scored using a visual scale where 1 = flat and 5 = tightly rolled leaves (O'toole et al. 1979) or by using a leaf rolling index which is defined as the ratio of width and projected area of the rolled leaves to the width of unrolled leaves (Matthews et al. 1990).

Adjustments for efficient photosynthetic activity in leaves are caused by the morphological diversity of leaf rolling (inward or outward) (Yuan et al. 2015). Leaf rolling slows down transpiration and enhances the accumulation of dry matter (Lang et al. 2004). Leaf rolling in the leaf blade is often due to lower water potential and turgidity of bulliform cells (Price et al. 1997). Moderate leaf rolling also ensures and maintains better water use efficiency compare to flattened and extremely rolled leaves (Juarez et al. 2004). Similarly, leaf rolling in wheat reduces the energy load on the leaf, lowers the surface temperature of the leaf, while still allowing the light to go deeper into the canopy which improves light interception and also reduces water loss (Rebetzke et al. 2001). Zhang et al. (2009a) also revealed that moderate leaf rolling increases photosynthesis and yield. Studies related to rolling and its association with wettability shows that inward and coiled-shaped leaves help in the channeling and movement of water droplets such as inward rolled leaves of *S. crassa* (Gürsoy et al. 2017b), twisting type leaves of *Stipagrostis sabulicola* (Roth-Nebelsick et al. 2012) and rolled leaf apex of xerophytic rosette families (Martorell and Ezcurra 2007). Therefore, leaf rolling is a significantly beneficial trait in wheat that can help to intercept atmospheric water towards root zone in a similar manner to *Stipagrostis sabulicola* spp. in the Namib Desert (Roth-Nebelsick et al. 2012). The architecture of leaf rolling dynamics of a wheat plant supports the high input use efficiency that can compensate for yield losses under drought stress.

#### Factors causing leaf rolling

Factors responsible for leaf rolling in plants are mostly caused by environmental factors such as water deficiency, temperature, and solar radiation. Other factors that are associated with leaf rolling include changes in photosynthetic rates, ion concentrations, alterations in the antioxidant systems, and cell formations.

##### Environmental factors

Water deficiency, increase in temperature, and solar radiation enhance leaf rolling in plants that increase drought resistance. Under limited water conditions, leaf rolling has a positive effect on plants by limiting water loss through stomata and leaf expansion (Ben-Amar et al. 2020). Bogale et al. (2011) revealed that optimum leaf rolling is also associated with more yield and high water productivity than loosely rolled one. Under high temperatures, leaf rolling is an important adaptation to curtail water losses. Heat stress also causes leaf rolling due to the reduction of cell size and number that leads to changing the leaf orientation and closure of stomata (Sarkar et al. 2021). Similarly, flag leaves of wheat plants roll their leaves under high temperatures that increase the water use efficiency (Kadioglu and Terzi 2007; Sarieva et al. 2010). For example, in wheat, leaves roll up on a sunny day from 11 a.m. to 2 p.m. (Kadioglu and Terzi 2007). Similarly, *A. villosum* plant also rolls their leaves upon exposure to sunlight that prevent photo-damage and thermal dissipation (Feng et al. 2002).

##### Change in ion concentration

Biochemical changes under a stress environment modify the ion concentration and cause leaf rolling in plants (Saglam et al. 2010). Studies related to ion concentration during leaf rolling are limited. Na^+^ and Ca^+2^ accumulated during leaf rolling both play an important role in water relations and the physiological process of a plant. The concentrations of K^+^ and Cl^−^ decreases during leaf rolling which inhibits the proper functioning of stomata (Saglam et al. 2010). During leaf rolling, malate and citrate accumulate in the vacuole and are involved in osmotic adjustment (Alvarez et al. 2008; Saglam et al. 2010).

##### Photosynthesis

Leaf rolling in plants affects CO2 and light use efficiency. Leaf orientation and rolling increase the photosynthetic efficiency (Zhang et al. 2009a; Sarieva et al. 2010). In C4 grass species, leaf rolling and its orientation induce stomatal and biochemical changes that increase photosynthetic capacity (Soares et al. 2008). Therefore, leaf rolling is an important trait for increasing photosynthesis in plants. Sarieva et al. (2010) recorded that the physiological role of leaf rolling increased water metabolism of leaves in wheat (Sarieva et al. 2010; Richards et al. 2010). Leaf rolling in wheat also prevented structural and functional damages. Under high temperature, leaf rolling is an adaptive response to increase pigmented and carotenoid contents in wheat (Luo et al. 2007; Kadioglu and Terzi 2007).

##### Alteration in antioxidant system

ROS (reactive oxygen species) are generated under stress conditions that involve destructive oxidative processes. Enzymatic antioxidant systems protect against toxic effects of ROS. Under drought stress conditions, antioxidant enzyme activities are enhanced as a result of leaf rolling (Smirnoff 2008). This might cause oxidative cross-linking and lignification of the cell wall (Saruhan et al. 2009, 2010). In *C. setosa*, changes in antioxidant enzyme activities were observed during leaf rolling (Terzi et al. 2009).

##### Cells involved in leaf rolling

In plants, cells that are responsible for leaf rolling are bulliform, hypodermal cells, and sclerenchyma cells (Kadioglu and Terzi 2007; Shi et al. 2007; Fujino et al. 2008). In the Gramineae species, specialized epidermal cells were present that are responsible for leaf rolling (Kadioglu and Terzi 2007). In some plant species, hypodermal cells located under the epidermis are also responsible for leaf rolling. For example, *C. setosa* exhibits huge hypodermal cells which cause leaf rolling when these cells shrink (Fujino et al. 2008). Sclerenchyma cells (lignified dead cells with secondary cell walls) also cause leaf rolling; for example, in rice, differential sclerenchyma cells cause inward rolling (Nelson et al. 2002).

The molecular mechanism of leaf rolling explained by Xu et al. (2018) revealed the final phenotype of leaf rolling is controlled by different genes that are involved in leaf polarity, the formation of bulliform cell and sclerenchyma cells, and cuticle development. Several genes responsible for leaf rolling have been identified from previous studies and are shown in Table 2. Finding these characteristics and their associated genes would also be beneficial in wheat.

**Table 2:** Studies available related to genes responsible for leaf rolling in cereals crops

Sr. No	Gene	Crop	Function	References
1	*RLD1 (rolled leaf1)*	Maize	Controls the upward rolled expression of leaf	Juarez et al. (2004)
2	*LBL1 (leafbladeless1)*	Maize	Determines the fate of adaxial cells	Canales et al. (2005)
3	*ADL1 (adaxialized leaf 1)*	Rice	Involved in rolling of leaves abaxially	Hibara et al. (2009)
4	*SLL1 (shallot-like 1)*	Rice	Regulates sclerenchyma cell development	Zhang et al. (2009a)
5	*OsAGO7 (argonatue)*	Rice	Forms miRNA effector complexes	Shi et al. (2007)
6	*RFS (rolled fine striped)*	Rice	Controls leaf polarity	Cho et al. (2018)
7	*CLD1 (curled leaf and dwarf 1)*	Rice	Controls cell wall integrity and osmotic homeostasis	Li et al. (2017)
8	*OsZHD1 (zinc finger homeodomain 1)*	Rice	Involved in abaxial curling	Xu et al. (2014)
9	*LC1 (leaf inclination 1)*	Rice	Regulates cell division	Zhao et al. (2013)
10	*RL14 (rolling leaf 14)*	Rice	Regulates secondary cell wall development	Fang et al. (2012)
11	*NRL1 (narrow and rolled leaf 1)*	Rice	Regulates cell formation	Hu et al. (2010)
12	*ROC5 (rice outer cell specific 5)*	Rice	Regulates bulliform cells formation	Zou et al. (2011)
13	*ACL1 (abaxially curled leaf 1)*	Rice	Determines leaf development	Li et al. (2010)
14	*REL1 (rolled and erect leaf 1)*	Rice	Positively regulates leaf rolling	Chen et al. (2015)
15	*REL2 (rolled and erect leaf 2)*	Rice	Determines leaf shape formation	Yang et al. (2016)
16	*OsLBD (lateral organ boundaries domain 3–7)*	Rice	Acts as a transcription activator	Pina et al. (2016)
17	*NAL7 (narrow leaf 7)*	Rice	Involved in leaf shape development by auxins	Fujino et al. (2008)
18	*OsI-BAK1 (brassinosteroi d insensitive 1- associated kinase 1)*	Rice	Involved in BR signaling pathway	Khew et al. (2015)
19	*OsARF18 (aux in response factor)*	Rice	Involved in Auxins signaling	Khew et al. (2015)
20	*SLL2 (shallot-like 2)*	Rice	Regulates of bulliform cell	Zhang et al. (2015)
21	*OsRRK1 (receptor-like cytoplasmic kinase 1)*	Rice	Determines the size and number of large specialized adaxial cells	Ma et al. (2017)
22	*CFL1 (curly flag leaf 1)*	Rice	Regulates cuticle development	Wu et al. (2011)

## Other leaf surface traits that have the potential for fog capturing in wheat

### Leaf erectness/angle

Leaf angle is defined as the inclination formed between the leaf blade and stem (Zhao et al. 2010; Mantilla-Perez and Salas Fernandez 2017). It is one of the hormonally regulated traits that is determined by collar tissues and their cell size (Zhao et al. 2010). The formation of leaf angle depends on the cell wall division, expansion, and composition in the lamina joint that helps to connect the leaf blade (Kong et al. 2017). Cytological observations in cereals reveal that the collar of erect leaves is due to the lack of elongation of longitudinal cells that results in the formation of a small inclination of the leaf with stem (Cao and Chen 1995; Hong et al. 2003; Zhao et al. 2010, 2013). An elongated cell of the collar of leaves causes the leaf blade to take a horizontal position (Hong et al. 2003; Zhao et al. 2013). Elongated cells are regulated by hormones including auxins, GA, and BRs. These hormones help to increase the relaxation of cell walls that ultimately increases flexibility and growth by the synthesis of polysaccharides (Luo et al. 2016).

To optimize photosynthetic machinery, leaf angle is one of the most important parameters in determining final grain yield. More optimal vertical redistribution of solar radiation throughout the canopy allows for denser planting of crops and enhances grain yield per hectare (Stewart et al. 2003). In cereals, hybrids that have an erect leaf angle show a 13% higher photosynthetic rate in the flag leaves at the heading time (Peng et al. 2008; Zhang et al. 2009b) and yielded 8–15% higher than common commercial cultivars (Zong et al. 2000). Cultivars/genotypes that exhibit erect and narrow type leaves often have higher photosynthesis rates, increased crop growth, and biomass production than horizontally positioned leaves (Kumagai et al. 2014). Rolled types of leaves in rice with erect leaf canopies increase the water use efficiency and yield by reducing the rate of transpiration and heat absorption (Zhang et al. 2009a, 2009b). Studies related to leaf erectness and its association with higher yield were reported in wheat, barley, and maize (Fischer and Edmeades 2010; Truong et al. 2015).

In wheat, many studies have conducted and reported that genotypes with narrow and erect type leaves had more leaf area for the exposure of sun rays, increased photosynthetic rate, superior carbon fixation, increased dry matter production, and higher final yield (Gardener et al. 1964; Parry et al. 2011). Leaf angle is one of the most important traits to determine the grain yield per hectare. Wheat ideotype has short stature and erect leaf to stem angle is ideal to achieve high yield potential (Donald 1968). Therefore, leaf angle could be considered a useful selection trait when breeding to increase wheat productivity and self-irrigation. Erect plant architecture also helps in the stem flow of droplets (Holder 2012). Studies related to steeper leaf angle and its association with wettability and channeling of droplets were reported in *S. sabulicola* (Namib grass) and *Phyllostachys aurea* (bamboo plant) (Roth-Nebelsick et al. 2012; Wigzell et al. 2016).

### Trichomes/prickle hairs on the leaf blade

Trichomes are fine out-growths on the leaf surface of the plant, also known as prickle hairs or appendages. They can be diverse in size, locality, density, morphology, and function. Trichomes are of two types—glandular or non-glandular (Werker 2000; Dada and Ohu 2013). Glandular trichomes release chemical compounds that act as a repellent against herbivores and pathogens while non-glandular act as a protective barrier under harsh environments by reflecting light, absorbing water and nutrients, and reducing transpiration (Mershon et al. 2015).

The expression of trichomes is influenced by environmental factors and their characteristics can change as a result of their environment during development (Hauser 2014). Phyto hormones such as gibberellin (GA), cytokinin (CK), salicylic acid (SA), jasmonic acid (JA), brassinolide (BR), and ethylene are also involved in the growth and development of trichomes on the leaf surface (Inthima et al. 2017; Traw and Bergelson 2003; Xia et al. 2018; Xiao et al. 2017; Zhou et al. 2013). For example, wheat density of hairs is controlled by cytokinin and jasmonic acid (Kobayashi et al. 2012).

During the early stages of plant development, the protective role of trichomes is high and then decreases gradually during the later stages of the plants’ life (Calixto et al. 2015). The plants’ chemical and structural properties are also changed and affected by shifting light regimes (Liakoura et al. 1997; Václavík et al. 2017). Leaves exposed to light radiation have a high density of trichomes and UV-absorbing capacity than shaded leaves (Fernández et al. 2014). Trichomes also play important role in disease resistance. Their presence on leaf surfaces prevents the contact and invasion of pathogen propagules (Gao et al. 2017; Rakha et al. 2017). Production of phenolic compounds released due to external stimuli helps in the inhibition of invading pathogen spores and their germination on the leaf surface (Beckman et al. 1972). Phenolic substances such as flavonoids play a role in the inhibition of fungal growth and spore formation (Yang et al. 1996).

Quantification of leaf pubescence was based on the pubescence index H. It is estimated by counting the trichome number and its length on the leaf surface (Pshenichnikova et al. 2017). In wheat, a study related to the association of pubescence with photosynthetic parameters revealed that poorly haired genotypes had more biomass than densely haired under optimal conditions (Pshenichnikova et al. 2019). Under drought conditions, trichome density increases whereas its length and photosynthetic parameters decrease (Pshenichnikova et al. 2019). The number and density of leaf hairiness vary due to environmental conditions; drought-resistant cultivar has more hairs than the humid environment (Krupnov and Tsapaikin 1990).

In grasses, leaf trichomes supply water to leaves because the basal part of the hairs is enriched with bulliform cells that absorb and store water (Aytasheva et al. 2006). Trichomes affect the water-leaf surface interactions by repelling water droplets toward the stem and soil thereby contributing to plant water uptake (Savé et al. 2000; Fernández et al. 2014; Konrad et al. 2015; Bickford 2016). It also increases water storage by limiting transpiration, modulating the energy balance, and reducing light absorbance (Savé et al. 2000; Fernández et al. 2014; Konrad et al. 2015; Bickford 2016). For example, *Opuntia microdasys* has moderate hair density that is involved in fog capturing and water retention (Ju et al. 2012). Similarly, *Stipagrostis sabulicola* also has dense hairs on leaf that helps in the self-irrigation by pinning and nucleation of fog water (Roth-Nebelsick et al. 2012). The physicochemical property of trichomes increases the leaf surface roughness and affects wetting capabilities (Fernández et al. 2014). In wheat, isogenic lines carrying an additional gene for dense leaf hairiness show that this trait significantly improves the water retention capacity in leaves (Likhenko 2007). Genes that determine the presence and density of trichomes in wheat were also found on chromosomes 7B and 7D respectively (Doroshkov et al. 2014). Trichomes are one of the important plant traits that should be considered when selecting for drought resistance in wheat.

### Grooves and channels on leaf sheath

In plants, grooves act as the channel for guided water flow and drawing water from the air (Malik et al. 2014). Role of grooves in the collection and transportation of atmospheric water is well-studied in some plant species. For example, *Stipagrostis sabulicola*, a grass species of Namib Desert, collects fog and utilizes it as an irrigation source (Roth-Nebelsick et al. 2012). This grass has involute-type leaves with grooves running alongside the axis of the leaf. Fog water is collected on the abaxial (lower) surface which becomes the outer surface due to the spiral curling of leaves. The droplets cling to the leaf surface and scattering rarely occurs. These droplets coalesce to form large pinned droplets of water that move downward towards the plant base (Roth-Nebelsick et al. 2012). Another type of grass, *Setaria viridis*, has grooves and a conical shape that helps in the directional transportation of water droplets (Xue et al. 2014). Similarly, studies on Bermuda grass, *Cynodon dactylon*, revealed that gradient grooves in the flattened surface of seed heads facilitate the directional movement of coalesced fog droplets (Sharma et al. 2016). Another investigation on *Dryopteris marginata* reported the importance of well-developed channels on the leaf surface that allows for efficient passage and transport of fog water. Grooves help to increase the water collecting rate (Sharma et al. 2018). The leaf surface of wheat has longitudinal grooves running throughout the lamina and a study revealed that medium to deep grooves help in the channeling and movement of water droplets (Hakeem et al. 2021). Therefore, longitudinal grooves on the wheat leaf surface are an important trait for the transportation of atmospheric water under stress.

## Conclusion and future prospectus

The exploitation of the wetting mechanism in wheat can increase the water status of plants by morphological features such as leaf rolling, leaf erectness, prickle hairs/trichomes, and longitudinal grooves. These morphological features can enhance drought resistance by alleviating the contradiction between water supply and demand. In wheat, morphological features may play an important role in water conservation and enhance leaf water potential that ultimately increases the photosynthesis rate and growth of the plant. Also, help the plant to keep its temperature low due to wettability and allow the maximum condensation of the atmospheric water on the wheat leaf surface.

Plants follow the two mechanisms of the wetting behavior of droplets, one of them is droplets of water that may be absorbed by leaf tissues and called foliar water uptake. While the second one is the movement of the droplets of water toward the base of the plant by stem or dripping off from the leaf tips. This information is revealed by the contact angle CA and hysteresis, either it is hydrophilic or hydrophobic.

Leaves of the wheat crop under water stress show the rolled type dynamics; its expression varies from tightly rolled to loosely rolled depending on the stress and environmental conditions. These rolling dynamics improve the overall efficiency of photosynthetic machinery increasing the source-to-sink translocation of assimilates that ultimately increase the biomass. The mechanism of leaf rolling explains that the expression of rolling shows due to the bulliform (large specialized adaxial epidermal cells), hypodermis, and sclerenchyma cells and also called water storage tissues. So rolled type phenotype in wheat helps the plant to store water by foliar uptake. Erect and spiral type phenotype of the plant may also help the plant in the directional movement of the remaining amount of water droplets toward the root zone without any disturbance of air forces. But optimization of leaf rolling dynamics under a foggy environment will be needed in wheat crops. Its impact on the water use efficiency and yield contributing parameters will also be needed. There is also a need to explore the expression of genes linked to the rolling dynamics for a detailed understanding of the mechanism.

Another trait that has potential in the atmospheric water acquisition in the wheat crop is groove and channels that run longitudinally throughout the leaf surface of the wheat plant. These grooves and channels may help in the directional movement of water droplets transportation. Indentation of grooves/channels on the leaf surface may provide the maximum adhesion of water droplets on the leaf surface and lead toward the elongation of a droplet in the flow direction. Grooves/channels also helped in the growth of larger droplets but their growth depends on the width and depth of grooves. They also provided the way like streams to atmospheric water. Similarly, the wheat plant also has a large variety of grooves from medium to deeper in width and depth ratio running throughout the leaf surface, which may help in the directional and guided movement of water droplets toward the root zone of the wheat plant.

The three-dimensional architecture of a leaf and its functionality depends on physiological processes such as photosynthesis, transpiration rate, and water use efficiency. It is logical to imagine that plants can also be engineered. Production of optimum size and shape of leaves help inefficient light-harvesting, water collection, and corresponding to faster growth rate and increased yield. Similarly, the leaf angle of the wheat plant may enhance the water use efficiency and directional movement of water toward the root zone/base of the plant without its dispersion. For this purpose, detailed optimization of leaf angle will be needed to check whether it is erect or semi-erect type canopy of wheat helped in maximum retention of water within root zone.

Trichomes/prickle hairs on the leaf surface also help in the reduction of transpiration rate and play a corresponding role in water conservation. Proximal and distal ends of the trichome change the wetting behavior of the droplets. In wheat, the presence and density of trichomes on the edges and leaf sheath especially distal ends of leaf blades may help in the inclination, collection, and transportation of water droplets but their optimization will be needed. Moreover, length and its density play an important role in physiological responses and drought resistance due to specific structure and its function. Further investigating the detailed ecophysiology, morphology, and molecular pathways of trichome in wheat may have theoretical and significant importance in plant breeding.

The exploitation of harvesting mechanisms in food crops is challenging as no previous studies have been conducted. Previously, few studies have been conducted on morphological adaptations but atmospheric water interception and its channeling, which depend on structural adaptations and external environment yet to be studied. There is a need to characterize the wheat germplasm and explore the morphological traits that can be utilized in this innovative mechanism. In the future, detailed optimization of the physical and chemical structure of wheat leaf surface traits for atmospheric water acquisition and its association with yield traits should be focused. According to wheat plant water requirements in different ecological regions, there is also a need to conduct studies related to water budget on the plant canopy and also leaf runoff to estimate the amount of water budget at critical irrigation stages. Optimization of wheat leaf surface structures for water collection has the potential to reduce the irrigation levels especially in arid regions where atmospheric water availability is more than groundwater. Moreover, molecular studies linked to the leaf surface traits should be conducted for the understanding of pathways and atmospheric water acquisition mechanisms. It can help to understand the regulation of the genes responsible for these traits. These, in combination with other approaches such as phonemics, will be the way forward to efficiently manipulate plant features for increasing yield and acquisition of atmospheric water in field crops.

## Data Availability

Not applicable.
